# The effect of fine particulate matter exposure on allergic rhinitis of adolescents aged 10–13 years: A cross-sectional study from Chongqing, China

**DOI:** 10.3389/fpubh.2022.921089

**Published:** 2022-10-25

**Authors:** Chunlan Qiu, Wei Feng, Xizhou An, Fangchao Liu, Fengchao Liang, Xian Tang, Ping Zhang, Xiaohua Liang

**Affiliations:** ^1^Department of Clinical Epidemiology and Biostatistics, Children's Hospital of Chongqing Medical University, National Clinical Research Center for Child Health and Disorders, Ministry of Education Key Laboratory of Child Development and Disorders, Chongqing Key Laboratory of Child Health and Nutrition, Chongqing, China; ^2^Department of Epidemiology, National Center for Cardiovascular Diseases, Fuwai Hospital, Chinese Academy of Medical Sciences and Peking Union Medical College, Beijing, China; ^3^School of Public Health and Emergency Management, Southern University of Science and Technology, Shenzhen, China

**Keywords:** allergic rhinitis, fine particulate matter, temperature, humidity, passive smoking, adolescents

## Abstract

**Background:**

Allergic rhinitis (AR) has become a tremendous disease burden worldwide. Only a few studies have explored the effects of environmental exposure on the prevalence of AR in children in China.

**Methods:**

In the present study, we investigated the associations of environmental exposure (including fine particulate matter (PM_2.5_), air humidity, temperature, and passive smoking) with AR in adolescents aged 10–13 years in Chongqing. Data from 4,146 participants in urban and rural areas between March 2019 and May 2019 were collected.

**Results:**

The overall prevalence of AR was 17.50% in adolescents. After adjusting for other covariates, AR was positively correlated with the annual mean PM_2.5_ concentration, monthly mean PM_2.5_ concentration and air temperature, and negatively related to air humidity. Furthermore, the annual mean PM_2.5_ was positively associated with the risk of AR after adjusting for air temperature and humidity. Passive smoking (PS) was marginally associated with a high risk of AR.

**Conclusion:**

High PM_2.5_ exposure, high air temperature, and low air humidity were associated with a high risk of AR in adolescents. Our findings have potential implications for public health strategies and interventions aimed at reducing the burden of AR in adolescents.

## Introduction

Allergic rhinitis (AR) affects 2–25% of children and has become a tremendous disease burden worldwide (1, 2). With the rapid progression of urbanization and the increase in westernized lifestyles, the incidence of AR in China has increased significantly, especially in urban areas. From 2010 to 2012, the China Children Homes Health (CCHH) study conducted in 10 cities in mainland China showed that the prevalence of AR varied from 24.0 to 50.8% in children (3). Patients with severe rhinitis symptoms might have a negative impact on quality of life (QOL), including sleep disorders, poor daytime activity, and memory decline, especially in adolescents (4, 5). Adolescence is considered to be a transitional period between childhood and adulthood and a critical stage for the prevention of atopic diseases due to immature immune function during this stage, which makes adolescents more susceptible to AR (6). Meanwhile, the onset of uncontrolled AR in adolescence may significantly affect the quality of life (QOL), and therefore, investigating the risk factors of AR is meaningful (5).

Previous studies indicated that genetic and environmental factors may play vital roles in the initiation of AR (7, 8). Only a few studies have explored the effects of environmental exposures on the prevalence of AR in Chinese children (9, 10), especially the effects of different types of air pollutants. PM2.5, also called fine particulate matter, is air pollutant particulate matter < 2.5 μm in aerodynamic diameter and is the most harmful pollutant among various air pollutants due to its high deposition rate in respiratory organs (11). PM_2.5_ pollution was the top concern in China in the last decade. According to the Environmental Quality Report (2015) released by the Ministry of Environmental Protection (MEP) (http://www.zhb.gov.cn/), the annual mean PM_2.5_ concentration in China was 50 μg/m^3^ (ranging from 11 to 125 μg/m^3^), which far exceeds the new National Ambient Air Quality Standard II (NAAQS-II, GB3095-R) of 35 μg/m^3^. A cross-sectional study found that higher air pollution levels were significantly associated with a greater risk for respiratory diseases and decreased lung function in Chinese children (12). On the other hand, exposure to passive smoking (PS) in childhood is common in Chinese families. However, limited studies evaluated the associations between PS and AR risk in China, and the conclusion was controversial. Only two cross-sectional studies conducted in kindergartens in Chongqing showed that half of the children were exposed to household PS (13). A previous study demonstrated that PS was a risk factor for AR (14). By contrast, a birth cohort study of 4,089 children indicated that early exposure to PS during embryonic stages or infancy was associated with an elevated risk of developing asthma up to adolescence but not with rhinitis or eczema (15).

Overall, the association between environmental exposure and AR in adolescents is under-investigated. To fill these gaps, this study hypothesizes that environmental exposure to air pollutants such as PM_2.5_ or passive smoking may have a potential impact on AR in adolescents. Thus, this study explored the associations of PM_2.5_, temperature, humidity, and PS with the risk of AR. To our knowledge, this is the first study to investigate the associations between atmosphere factors and AR in adolescents in Southwest China, which experiences extremely humid weather.

## Methods

### Study design and participants

**Figure 1:**
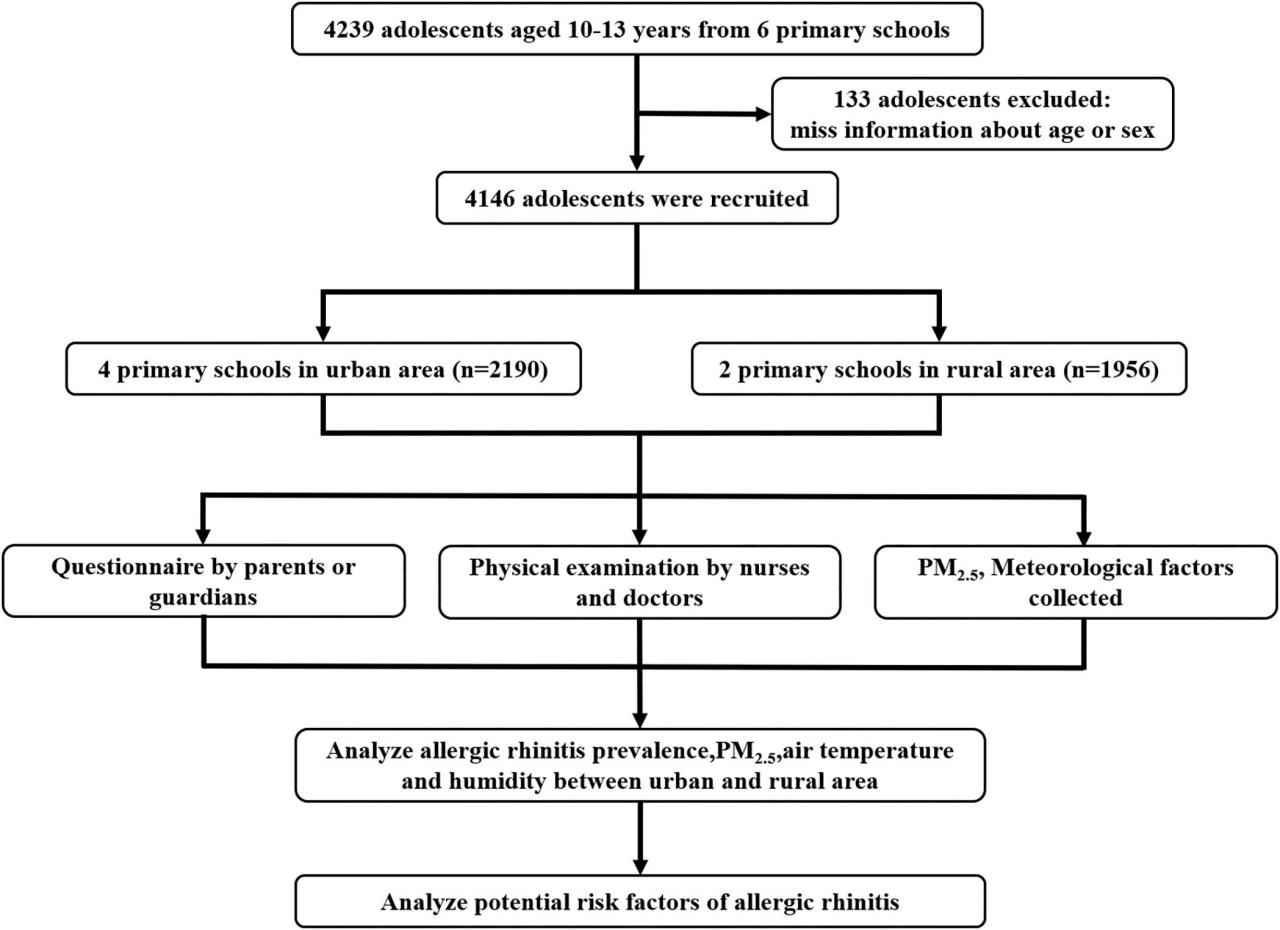
Flowchart of the study.

This cross-sectional study was carried out in Chongqing, from March to May 2019 (Figure 1). A stratified cluster sampling comprising two stages was used to include participants from two counties in Chongqing that represent urban and rural areas, respectively; then, two communities per county were randomly selected, and all adolescents recruited in this cohort were from grade 5 and grade 6 in elementary schools. The adolescents were included if they met the following criteria: children (1) aged between 10 and 13 years; (2) who resided in the selected communities for more than 6 months; and (3) who had consent provided by the parents and children for participation. The exclusive criteria were as follows: children (1) who cannot accept the physical examination during the study period and (2) who have serious diseases (e.g., nephropathy, bronchopulmonary dysplasia, cardiovascular disease, or cancer). The sample size was calculated with the following parameters: an α level of 0.05, a power of 95%, a prevalence of AR components of 13%, and a prevalence in the population of 10%, using the formula $n=\frac{{\left(Z_{1-\alpha /2}\;+\;Z_{1-\beta }\right)}^{2}[(p_{1}(1-p_{1})\;+\;p_{2}(1-p_{2})]}{\delta^{2}}$. Assuming an attrition rate of 20%, at least 4,133 participants were needed. Finally, 4,279 adolescents were invited, and 4,146 participants were included in this study, with a response rate of 96.89%. The Institutional Review Board at the Children's Hospital of Chongqing Medical University provided the approval for this study (No. 2019-86). Informed consent was provided by both the adolescents and their parents/guardians.

### Demographic variables

Demographic information was collected using a questionnaire. Socioeconomic variables included parental occupation, paternal education level, household income, marital status of parents, and family structure. The parental education level was measured on a four-point scale, ranging from less than high school to postgraduate education. The demographic questionnaire was completed by the parents/guardians of the adolescents after standard training by the research group.

### Physical examination

Anthropometric measurements were conducted by well-trained pediatric nurses. Well-trained nurses from the Children's Hospital of Chongqing Medical University carried out the physical examinations. After they were rigorously trained, an examination based on the operation manual was carried out, and the training was according to the anthropometric examination guidelines recommended by the World Health Organization (WHO) (16). All anthropometric examinations strictly followed the operation manual during the physical examinations in this study. In addition, an automatic ultrasonic detector (Ws-h300d, Shanghai Woshen Inc., Shanghai, China) was used for height and weight measurements. Waist circumference (WC) was measured two times for children who fasted, wore close-fitting clothing, stood upright, and relaxed the abdomen. The measurements were taken at the center of the navel at the end of exhalation without inhalation and averaged. The body mass index (BMI) was calculated as the ratio of weight in kilograms to height in meters squared (kg/m^2^).

### Diagnostic criteria

The diagnosis of current AR followed the clinical practice guideline of allergic rhinitis (17). The diagnosis was made based on the self-report of nasal symptoms (i.e., sneezing, runny nose, nasal itching, and nasal congestion, without a cold or the flu) and eye involvement in the past 1 month, combined with diagnosis confirmation by pediatricians during the physical examination at school. We did not perform atopy tests such as skin prick tests (SPTs) or serum allergen-specific IgE (sIgE) tests or nasal allergen challenge (NAC). Passive smoking was defined as the presence of at least one family member who smoked at least five cigarettes in the past 30 days at home during the survey (18).

### PM_2.5_ exposure assessment

Long-term exposure levels of PM_2.5_ for each participant were assessed using satellite-based PM_2.5_ concentrations at 1-km spatial resolution. Monthly mean PM_2.5_ concentrations across China were estimated from 2018 to 2019 using machine learning approaches (19). To assess individual long-term exposure levels of PM_2.5_, residential addresses collected at baseline and follow-up visits were geocoded for each participant over the entire study period. Monthly mean PM_2.5_ concentrations were assigned to the participants who were at risk for AR during the follow-up period, based on the grid cells in which they resided. For those who changed their residential addresses during the survey, average monthly mean PM_2.5_ concentrations between the previous address and the current address were used. The annual average PM_2.5_ was calculated by the sum of the monthly average values of the current year divided by exposure months.

### Air temperature and relative humidity data

We obtained the monthly air temperature and humidity data from European Center for Medium-Range Weather Forecasts atmospheric reanalysis of the global climate version 5 data set. The calculation of air temperature and humidity for children who changed their addresses was similar to those of PM_2.5_.

### Statistical analyses

Differences in anthropometric variables between the two groups were assessed using Student's *t*-test. Continuous variables that did not satisfy the normal distribution were presented as median (P_25_, P_75_), and the Wilcoxon rank sum test was used for comparison of these variables between the two groups. The prevalence rates of AR were reported as numbers (*n*) and percentages of the total (%), and the χ^2^ test was used to test the difference between the two groups. Moreover, univariate logistic regression (model 1: crude model) was performed to investigate the association between PM_2.5_ and AR risk. Then, age, sex, BMI, PS, family income, parental marital status, paternal education level, and mother's education level were adjusted for multivariate logistic regression in model 2. All statistical analysis was performed by SAS 9.4 software (Copyright © 2016 SAS Institute Inc. Cary, NC, USA). A significant difference was defined by an α level of 0.05 with a two-sided test.

## Results

### General characteristics of the participants

The general characteristics of the participants are shown in Table 1. The mean age was 11.74 ± 0.71 years, and 51.64% were male. The prevalence of adolescent AR was 17.50%. Between the two groups of adolescents with and without AR, age, height, and waist circumference were significantly different. Mother's and father's education levels were significantly correlated with the incidence of AR. The total prevalence of PS was 40.69%, and it was significantly related to AR incidence (*P* = 0.0022).

**Table 1:** Characteristics of study participants.

**Variables**	**Total number** **(*n* = 4,146)**	**Non-rhinitis group** **(*n* = 3,439)**	**Rhinitis group** **(*n* = 707)**	**F**	***P***
Age, year	11.74 ± 0.71	11.75 ± 0.72	11.69 ± 0.64	4.35	0.0371
Male, *n* (%)	2,141 (51.64)	1,713 (51.40)	374 (52.90)	0.5286	0.4672
**Anthropometric measures**					
Height, cm	151.35 ± 7.88	151.23 ± 7.93	152.15 ± 7.80	7.12	0.0077
Weight, Kg	43.54 ± 10.47	43.45 ± 10.40	44.15 ± 10.77	2.39	0.1225
Waist, cm	64.51 ± 10.00	64.37 ± 9.95	65.44 ± 10.23	6.08	0.0138
BMI, Kg/m^2^	18.84 ± 3.49	18.83 ± 3.43	18.93 ± 3.78	0.43	0.5124
**Socioeconomic measures**					
Income, Yuan/year					
~100,000	2,069 (65.27%)	1,673 (65.81%)	343 (61.80%)	3.2283	0.1991
~200,000	764 (24.10%)	602 (23.68%)	147 (26.49%)		
>200,000	337 (10.63%)	267 (10.50%)	65 (11.71%)		
Marriage status					
Double parents	2,922 (83.82%)	2,326 (83.28%)	527 (86.25%)	3.2649	0.0708
Single parents	564 (16.18%)	467(16.72%)	84(13.75%)		
Mother's education, y					
~9	1,280 (37.31%)	1,062 (38.73%)	183 (30.15%)	22.4523	< 0.001
~12	1,162 (33.87%)	930 (33.92%)	206 (33.94%)		
≥15	989(28.83%)	750 (27.35%)	218 (35.91%)		
Father's education, y					
~9	1,015 (30.10%)	831 (30.80%)	154 (25.88%)	14.6567	0.0007
~12	1,220(36.18%)	992 (36.77%)	200 (33.61%)		
≥15	1,137 (33.72%)	875 (32.43%)	241 (40.50%)		
Passive smoking	1,392 (40.69%)	1,082 (39.47%)	278 (46.26%)	9.3931	0.0022

### PM_2.5_, air temperature, and humidity associated with AR

The annual mean PM_2.5_ concentration, monthly mean PM_2.5_ concentration, and air temperature in the AR group were significantly higher than those of in the non-rhinitis group, while the annual mean and monthly mean air humidity in the AR group were significantly lower than those in the non-rhinitis group (all *P* < 0.05, Table 2).

**Table 2:** PM_2.5_, air temperature, and humidity in the allergic rhinitis and non-rhinitis groups.

**Variables**	**Non-rhinitis group** **(*n* = 3,439)**	**Rhinitis group** **(*n* = 707)**	**F**	***P***
Annual mean PM_2.5_, μg/m^3^	44.44 ± 2.46	45.15 ± 2.14	50.44	< 0.001
Monthly mean PM_2.5_, μg/m^3^	38.33 ± 3.86	39.08 ± 3.45	22.77	< 0.001
Annual mean RH, %	77.09 ± 1.77	76.73 ± 1.52	25.93	< 0.001
Monthly mean RH, %	72.04 ± 1.64	71.84 ± 1.36	9.48	0.002
Annual mean temperature, °C	17.48 ± 0.90	17.66 ± 0.76	24.81	< 0.001
Monthly mean temperature, °C	20.64 ± 1.34	20.89 ± 1.14	21.33	< 0.001

PM2.5: fine particulate matter; RH: relative humidity.

A logistic regression model was used to analyze the potential risk factors of AR. The risk of AR was significantly positively associated with the annual mean PM_2.5_ concentration (OR: 1.110, 95% CI: 1.054, 1.169, *P* < 0.001), monthly mean PM_2.5_ concentration (OR: 1.032, 95% CI: 1.001, 1.064, *P* = 0.041), annual mean air temperature (OR: 1.190, 95% CI: 1.035, 1.369, *P* = 0.015), and monthly mean air temperature (OR: 1.102, 95% CI: 1.006, 1.207, *P* = 0.038) but negatively related to annual mean air humidity (OR: 0.915, 95% CI: 0.854, 0.981, *P* = 0.013), after adjusting for demographic and other covariates (Table 3).

**Table 3:** ORs and 95% CI of environmental exposure for allergic rhinitis.

**Variables**	**β**	**ORs (95%CIs)**	***P***
Annual mean PM${}_{2.5}^{*}$, μg/m^3^	0.026	1.110 (1.054, 1.169)	< 0.001
Monthly mean PM${}_{2.5}^{*}$, μg/m^3^	0.016	1.032 (1.001, 1.064)	0.0412
Annual mean air humidity^*^, %	0.036	0.915 (0.854, 0.981)	0.0127
Monthly mean air humidity^*^, %	0.036	0.967 (0.901, 1.038)	0.3517
Annual mean air temperature^*^, °C	0.0713	1.190 (1.035, 1.369)	0.0145
Monthly mean air temperature^*^, °C	0.0466	1.102 (1.006, 1.207)	0.0377

* Adjusted for age, gender, BMI, passive smoking, annual family income, parental marital status, mother's education level, father's education level, and region.

Furthermore, the association between the PM_2.5_ exposure level by tertiles and the risk of AR was analyzed by logistic regression analysis (Table 4). Similarly, compared with the tertile 1 (T1) level, participants with annual mean PM_2.5_ and monthly mean PM_2.5_ exposure in the T3 level had a higher odds for AR risk (annual mean PM_2.5_: adjusted OR: 1.806, 95% CI: 1.364–2.391, *P* = 0.004; monthly mean PM_2.5_: adjusted OR: 1.497, 95% CI: 1.151–1.948, *P* = 0.002).

**Table 4:** Logistic regression analysis of PM_2.5_ exposure level with allergic rhinitis risk.

**Model**	**Comparative relationship**	**ORs (95%CIs)**	***P***
**Annual mean PM**${\;}_{2.5}^{*}$, **μg/m**^**3**^			
	T2 vs. T1	1.496 (1.140,1.963)	0.3083
	T3 vs. T1	1.806 (1.364,2.391)	0.0004
**Monthly mean PM**${\;}_{2.5}^{*}$, **μg/m**^**3**^			
	T2 vs. T1	1.137 (0.867,1.492)	0.5095
	T3 vs. T1	1.497 (1.151,1.948)	0.0015

*Adjusted for age, gender, BMI, passive smoking, annual family income, parental marital status, mother's education level, father's education level, and region.

T, tertile.

### The risk factors of AR using a multivariate logistic regression model

Annual mean PM_2.5_ was positively associated with the risk of AR after adjusting for air temperature and humidity (OR: 1.187, 95% CI: 1.090–1.293, *P* < 0.001). PS was marginally associated with a high risk of AR (OR: 1.214, 95% CI: 0.998–1.478, *P* = 0.050). Also, a higher mother's education level (OR: 1.231, 95% CI: 1.041–1.456, *P* = 0.015) was associated with a high risk of AR (Table 5).

**Table 5:** A multivariate logistic regression model analyzed the association of PM_2.5_ with allergic rhinitis risk.

**Variables**	**ORs (95%CIs)**	***P***
Model 1: Annual mean PM_2.5_		
Father's education level	0.965 (0.813, 1.146)	0.685
Mother's education level	1.231 (1.041, 1.456)	0.015
Passive smoking, (Yes vs, No)	1.214 (0.998, 1.478)	0.052
Annual mean air humidity, %	0.956 (0.743, 1.230)	0.726
Annual mean air temperature, °C	0.780 (0.464, 1.312)	0.348
Annual mean PM_2.5_, μg/m^3^	1.187 (1.090, 1.293)	< 0.001
Model 2: Monthly mean PM_2.5_		
Father's education level	0.961 (0.809, 1.142)	0.653
Mother's education level	1.235 (1.044, 1.461)	0.013
Passive smoking, (Yes vs. No)	1.214 (0.998, 1.477)	0.052
Annual mean air humidity, %	2.021 (1.497, 2.730)	< 0.001
Annual mean air temperature, °C	2.614 (1.761, 3.880)	< 0.001
Monthly mean PM_2.5_, μg/m^3^	1.003 (0.956, 1.053)	0.888

## Discussion

To our best knowledge, this is the first large-scale epidemiological survey in Southwest China to investigate the association of external environmental pollutant exposure with AR risk in adolescents in China. This study provided further evidence that PM_2.5_ exposure, air temperature, and air humidity were associated with a high risk of adolescent AR.

The International Study of Asthma and Allergies in Childhood (ISAAC) Phase Three found that current AR in the 13- to 14-year age group was 14.6%, which was consistent with our results (20). The prevalence of AR in Chongqing was lower than that reported in previous epidemiological studies in China (21, 22). The difference may be explained by the difference in age range, sampling method, and diagnostic criteria. Other studies focused on the prevalence of AR in preschoolers (23, 24), but the participants in our study were adolescents aged 10–13 years. Moreover, the participants in our study were recruited from communities, which was more representative than some other studies only including routine follow-up patients from hospitals (25). In addition, most of the epidemiological studies used self-reported AR based on the ISAAC questionnaire to make the diagnosis (26, 27); however, a recent study conducted on Korean children showed that the accuracy of AR diagnosis based on the ISAAC questionnaire is about 60% (28). In this study, AR was diagnosed based on a questionnaire and pediatrician confirmation.

China had the highest PM_2.5_ concentration worldwide. In 2013, only 4.1% of Chinese cities achieved the annual mean standard of ≤ 35 μg/m^3^ (29). The annual mean PM_2.5_ concentration in urban Chongqing in 2012–2013 was 75.4 ± 42.2 μg/m^3^ (30). During the study period, the annual mean PM_2.5_ concentration in Chongqing was much lower than that before, which might contribute to the decreased prevalence of AR in this study compared to previous publications from Chongqing (31). However, our study showed that the PM_2.5_ concentration in urban areas of Chongqing still exceeded the WHO Interim Target I (IT-1) and second-level criterion of the Chinese national air quality standard (35 μg/m^3^).

The association between PM_2.5_ and AR risk remains inconsistent. In a pooled analysis of six birth cohorts, point estimates for associations between nitrogen dioxide, PM_2.5_ mass, and PM_2.5_ absorbance with AR were positive, but only PM_2.5_ was statistically significant (32). However, a longitudinal study that followed up children from their birth to 10 years found that PM_2.5_ did not increase the prevalence of doctor-diagnosed AR (33). While a school-based survey on allergic diseases in kindergarten children in Taipei found that exposure to PM_2.5_ might increase the risk of AR (34). Our study showed that the PM_2.5_ exposure level was positively associated with AR risk in adolescents, which was consistent with another study (35). However, the age of the participants in our study differed from previous studies. Norbäck et al. showed that PM_2.5_ exposure could also affect the respiratory health of children aged 3–6 years (36), which suggests a possibility that PM_2.5_ exposure could also affect the respiratory health for a long time from childhood to adolescence. Furthermore, the definitions were not identical between this survey and other studies (37).

Changes in meteorological factors can lead to changes in allergens such as pollen, which can induce or aggravate the symptoms of AR (38, 39). The results of this study showed that the risk of AR incidence elevated with the increase in air temperature in spring. However, another study provided evidence of a negative effect of abrupt temperature drops between two adjacent days on childhood AR (40). Further investigations to compare the effects of climate change on AR in different seasons might be needed. Previous studies elucidated that a decrease in air humidity may reduce the threshold of nasal mucosa response to an allergic reaction and increase the incidence of AR (41, 42), which could explain our findings. Another analysis of respiratory symptom scores of patients with AR from Shanghai showed that both temperature and humidity are negatively correlated with subjective symptom scores, showing that the symptom scores increase by 0.04 points for every 1°C decrease in temperature or 10% decrease in humidity (25). However, they only analyzed the correlations between monthly data on the subjective symptoms of 351 children who were previously diagnosed with AR. The difference between this study and our study might be due to the phenological changes during the season span, different participants' chosen methods, and the power of sample size.

Socioeconomic factors including mother's education level have been identified as a risk factor for allergic diseases (43). Mother's education has been found to be a risk factor for atopic diseases like rhinitis, allergies, and eczema, which consistently followed reverse socioeconomic gradients, being more common among advantaged children (44). This situation may be explained by the more frequent exposure to microorganisms among children of low social status. Also, the hygiene hypothesis (45)—growing up in cleaner environments, which is more common in more advantaged households, might compromise the development of a child's immune system—could be another explanation for this phenomenon between allergic diseases and mother's education level. In the present study, we noticed mothers' education level was positively related to the incidence of AR after adjustment for other factors, which was consistent with previous reports. However, in addition to the reason previously described, the urban–rural difference and cultural factors must be taken into consideration. In this cohort, the maternal education level and other related factors such as PM2.5, humidity, and temperature were significantly different between urban and rural districts, which suggested totally different socio-economical and environmental conditions between urban and rural areas, which might enhance the effect of maternal education difference. Also, the culture where mothers spent more time taking care of children in most Chinese families might strengthen the effect of the maternal factor on the children's health as in this study (46); hence, the mother's educational level was significantly related to the incidence of AR, rather than the father's educational level.

Self-reported PS was an important risk factor for adolescent AR, with nearly half of the participants self-reporting PS at a relatively young age (47). Data from 68 low- and middle-income countries showed that the overall prevalence of PS in young adolescents aged 12–15 years was 55.9%, ranging from 16.4% in Tajikistan to 85.4% in Indonesia (48). Despite the relationship between lower airway diseases and PS being documented, there were limited data elucidating the relationship between PS and AR risk (49), and the conclusion was controversial. A study from the United States reported that after multivariate adjustment, an increased incidence rate of self-reported rhinitis was observed in children and adolescents (50). Foliaki et al. found that PS, with two or more people smoking in the house, was not associated with AR (51). On the contrary, Gonzalez-Diaz et al. showed that in both 6- to 7-year-old and 13- to 14-year-old children, AR was associated with PS at home (52). The latest systematic review and meta-analysis demonstrated that in children and adolescents, AR was associated with both active smoking and PS (14). In line with previous studies, our study also found that there was marginal statistical significance between PS and AR in adolescents. The WHO requests countries to ban smoking in enclosed public places, but reducing PS for children at home is also critical, which might mainly rely on non-regulatory measurements, including education and clean air social norms (14).

There are several strengths to this study. First, to our knowledge, the present study is the first to investigate the effect of both the long- and short-term environmental exposure of PM_2.5_, air temperature, and air humidity on the incidence of AR in healthy adolescents in two counties in the community in Chongqing, and the results suggested that long-term PM_2.5_ exposure was significantly correlated with the incidence of AR. Second, this study included a large sample both from urban and rural areas; therefore, the result may represent the adolescents in Chongqing. Third, the data used in this study were from a cohort study, and the long-term dose exposure effect of environmental pollution was identified, and the results were validated as major variables were collected both at baseline and at follow-up.

## Limitations

The present study has several limitations. First, this study is a cross-sectional study. Longitudinal analysis is required to study the incidence of AR, and the association between risk factors and AR incidence in the four seasons. Second, information on allergic sensitization or inflammatory cytokine levels was undetected for further confirmation of the diagnosis of AR. Our study has focused on the combination of nasal and conjunctival symptoms (in the absence of intercurrent infection) and a pediatrician's physical examination as the most relevant definition of AR. This may lead to a few overdiagnosis of AR since we did not perform atopy tests, including SPTs and sIgE tests and NAC, to confirm the allergen of the children with rhinitis. Third, we only analyzed the concentrations of PM_2.5_ but did not analyze the different components of PM_2.5_ (iron, copper, nickel, zinc, vanadium, sulfur, potassium, and silicon). PM components, in particular iron, copper, and zinc, reflect poorly regulated non-tailpipe road traffic emissions, which may increase the risk of AR in children. Future studies should analyze the effect of the concentration and composition of different PM_2.5_ on AR risk.

## Conclusion

In conclusion, this study partially filled in the gap in the epidemiological investigation of AR in Chinese adolescents. High PM_2.5_ exposure, high air temperature, and low air humidity were associated with a high risk of AR in adolescents, and PS was associated with a higher risk of AR.

## Data availability statement

The raw data supporting the conclusions of this article will be made available by the authors, without undue reservation.

## Ethics statement

The studies involving human participants were reviewed and approved by the Institutional Review Board at the Children's Hospital of Chongqing Medical University. Written informed consent to participate in this study was provided by the participants' legal guardian/next of kin.

## Author contributions

XL conceived and designed the study. FLia, FLiu, and XA collected the data. XL, CQ, PZ, WF, and XT participated in the physical measurement. CQ wrote the manuscript. All authors critically reviewed and approved the final manuscript.

## Funding

This work was supported by the National Key Research and Development Project of the Ministry of Science and Technology of China (2017YFC0211705), the Major Health Project of Chongqing Science and Technology Bureau (No. CSTC2021jscx-gksb-N0001), CQMU Program for Youth Innovation in Future Medicine (No. W0088), Young and Middle-aged Medical Outstanding Expert Project of Chongqing Municipal Health Commission, Joint Medical Research Project of Chongqing Municipal Health Commission and Chongqing Science and Technology Bureau (2020MSXM062), Technology Foresight and Institutional Innovation Project of Chongqing Science and Technology Bureau (cstc2020jsyj-zzysbAX0016), Young Scientists Fund Program of the National Natural Science Foundation of China (81502826), and Young Scientists Fund Program of the Education Commission of Chongqing (KJQN201900443). The funders had no role in the study design, the data collection and analysis, the decision to publish, or the preparation of the manuscript.

## Conflict of interest

The authors declare that the research was conducted in the absence of any commercial or financial relationships that could be construed as a potential conflict of interest.

## Publisher's note

All claims expressed in this article are solely those of the authors and do not necessarily represent those of their affiliated organizations, or those of the publisher, the editors and the reviewers. Any product that may be evaluated in this article, or claim that may be made by its manufacturer, is not guaranteed or endorsed by the publisher.
